# Acute effect of smoking on plasma Obestatin levels

**DOI:** 10.1186/1617-9625-8-2

**Published:** 2010-01-07

**Authors:** Asterios Kukuvitis, Marios Froudarakis, Stavros Tryfon, Argyris Tzouvelekis, Maria Saroglou, Nikolaos Karkavitsas, Demosthenes Bouros

**Affiliations:** 1 Department of Endocrinology, Medical School Democritus University of Thrace, Alexandroupolis, Greece; 2 Department of Pneumonology, Medical School Democritus University of Thrace, Alexandroupolis, Greece; 3 1st Department of Pneumonology, GH "G Papanikolaou", Thessaloniki, Greece; 4 Department of Nuclear Medicine, Medical School University of Crete, Heraklion, Greece

## Abstract

**Background:**

Smoking and smoking cessation are considered to be associated with weight changes. We have recently shown that smoking acutely increases plasma levels of ghrelin, a known orexigenic hormone.

Obestatin is a peptide encoded by the ghrelin gene, which opposes ghrelin effects on food intake. We conducted a study in adult volunteers measuring plasma levels of obestatin immediately after initiation of smoking.

**Methods:**

31 volunteers (mean age 32.2 ± 9.2 years and mean BMI 25.7 ± 4.1), 17 smokers and 14 non-smokers, were enrolled in our study. The 2 groups were matched in age and BMI. Plasma obestatin concentrations were determined at baseline (T0), 2 (T2), 5 (T5), 15 (T15), and 60 (T60) minutes after the initiation of smoking.

**Results:**

In all 31 subjects, no significant difference in the mean values of plasma obestatin levels was observed from baseline at T2, T5, T15 and T60 after initiation of smoking (overall p = 0.15). However, a trend for higher obestatin levels was noted in smokers vs non-smokers (overall p = 0.069), which was not related to the pack-years.

**Conclusion:**

On the contrary with ghrelin's response after smoking initiation, there is no such an acute response of plasma obestatin levels.

## Background

Smoking and smoking cessation are considered to be associated with weight changes [1]. Weight gain is a primary reason for not trying to quit smoking. The mechanism of weight gain includes decreased resting metabolic rate, increased energy intake, decreased physical activity and increased lipoprotein lipase activity [2]. We have recently shown that smoking acutely increases plasma levels of ghrelin, a known orexigenic hormone [3]. This finding was unexpected in terms of explaining the known anorectic effect of smoking [1].

Obestatin is a peptide encoded by the ghrelin gene, which opposes ghrelin effects on food intake [4]. Treatment of rats with obestatin decreased body - weight gain. We hypothesized that smoking might acutely increase plasma levels of obestatin in addition to ghrelin levels. Therefore we conducted a study in adult volunteers measuring plasma levels of obestatin immediately after initiation of smoking.

## Methods

Thirty one volunteers, 17 smokers and 14 non smokers, were enrolled in our study (Table 1). The subjects received both oral and written information before they gave their written consent to participate. The study was approved by the Ethics Committee of the University Hospital of Alexandroupolis. None of the individuals had a history of gastric operation or any other serious health problem. Routine laboratory testing was performed to all of them.

**Table 1:** Demographic characteristics of all 31 subjects

	*n*	*Age (years)*	*BMI (kg/m*^2^)
**All subjects**	31	32.2 ± 9.2	25.7 ± 4.1
**Males**	23 (74.2%)	31.8 ± 8.8	26.3 ± 3.7
**Females**	8 (25.8%)	33.3 ± 10.6	23.7 ± 5
**Smokers**	17 (54.8%)	29.4 ± 7.8	26.7 ± 3.7
**Non-smokers**	14 (48.2%)	27.4 ± 8.6	24.2 ± 3.7

Values are expressed as mean ± standard deviation (SD).

Blood samples were collected after overnight fasting. After baseline measurements, the participants smoked one filtered cigarette containing 0.8 mg of nicotine under standardized conditions: every 15 seconds, a puff lasting 5 seconds was taken, and the whole cigarette had to be smoked within 5 minutes. Plasma obestatin concentrations were determined at baseline (T0), 2 (T2), 5 (T5), 15 (T15), and 60 (T60) minutes after the initiation of smoking.

Plasma obestatin levels were measured with a commercial radioimmunoassay (RIA) kit (Phoenix Pharmaceuticals, Belmont, CA) using a 125I-labeled obestatin as a tracer and a polyclonal antibody rose in rabbits against human obestatin. No cross-reactivity was found with human ghrelin, motilin, leptin or other relevant molecules. All samples were run in duplicate, and mean values were used for subsequent analysis. The sensitivity of the assay was 215.5 pg/ml. Intra- and inter-assay coefficients of variation (CV) reported by the manufacturer were < 5% and < 12% respectively. The linear range was 50-6400 pg/ml. Plasma was frozen in aliquots at -800 C immediately after centrifugation (40 C, 1600 g for 15 min).

### Statistical analysis

The mean values and standard deviation (SD) for the studied groups were compared using the Student's t-test both paired (comparison between 2 groups) and unpaired (factorial effect on groups). The chi squared test was used to determine whether a difference existed between the demographic parameters studied (smoking, gender). Also simple regression analysis was performed to check for possible relationships between demographic parameters and obestatin values. Significance between groups was set at p = 0.05. Statistical evaluation of the data was done using StatViewTM 4.5 software (Abacus Concepts Inc., Berkeley Ca.).

## Results

We evaluated the plasma levels of obestatin in 31 subjects, 23 males (74.2%) and 8 females. Demographic characteristics of all subjects are shown in Table 1. No significant difference in age was noted between male and female subjects (p = 0.68) or between smokers and non smokers (p = 0.54). Also, no significant difference in BMI was observed between males and females (p = 0.13) and between smokers and non smokers (p = 0.11). No significant relationship was found between gender and smoking (chi square = 0.28, p = 0.59), and the mean pack-years between male (18.4 ± 9.9) and female (11.2 ± 9.4) smokers was not significant different (p = 0.21).

**Figure 1:**
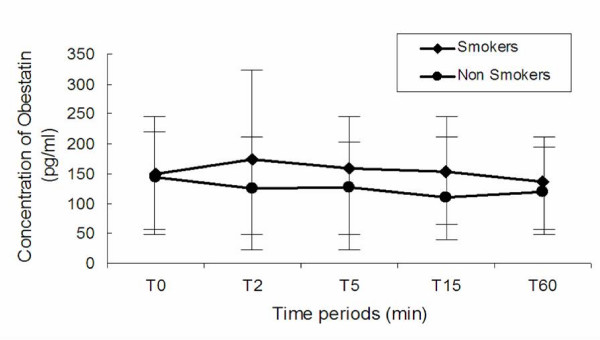
**Effect of smoking on obestatin levels for 17 subjects smokers (red line) and for 14 subjects non-smokers (blue line)**. Error bars express standard deviation.

The mean values of plasma obestatin levels for all 31 patients were at baseline T0 = 148.2 ± 96.8 pg/ml, at 2 minutes T2 = 157.3 ± 133 pg/ml, at 5 minutes T5 = 147.7 ± 100.2 pg/ml, at 15 minutes T15 = 141.6 ± 98.4 pg/ml and at 60 minutes T60 = 127.7 ± 85 pg/ml. No significant difference was observed from baseline at T2, T5, T15 and T60 (overall p = 0.15). The mean values between smokers and non smokers were not significant different at any time, although there is a trend (overall p = 0.069) of more acute onset of obestatin levels in smokers, after acute smoking (Figure 1). The increase of serum levels of obestatin at any time was not related to the number of pack-years. Also BMI (r2 = 0.32, p = 0.37), or age (r2 = 0.065, p = 0.21) were not factors influencing the serum levels of obestatin. No significant difference (p = 0.58) was observed at baseline levels of obestatin between males (mean: 155.8 ± 90 pg/ml) and females (mean: 127.6 ± 118.7 pg/ml) and gender was not a factor influencing the serum levels of obestatin at any time (overall p = 0.82).

## Discussion

We sought to find if an acute response of obestatin exists after initiation of smoking similar of that of ghrelin [3]. To our knowledge, this is the first study reporting results of plasma obestatin after smoking in volunteers. No acute effect of smoking was noticed. Taking under consideration the ghrelin increase [3], we speculate that obestatin does not contribute to the anorexic effect of smoking.

Obestatin (a ghrelin-associated peptide) is a 23-amino acid peptide that is derived from post-translational processing of the preproghrelin gene. Mutant mice with a deletion of the ghrelin gene did not show impaired appetite [5,6] probably because these animals lacked both orexigenic ghrelin and anorexigenic obestatin. Also in knockout mice, the lack of the orexigenic signal exerted by ghrelin may be counterbalanced by the concurrent absence of the anorexigenic signal exerted by obestatin [4]. Initially it has been thought that obestatin binds to the orphan G protein-coupled receptor 39 (GPR39), although this has been recently questioned [5]. In addition obestatin has been found to have little or no effect on pituitary hormone axis, contrary to ghrelin action [6].

Although the difference of obestatin levels between smokers and non-smokers in our study was not significant, we observed a trend for higher levels of obestatin in smokers when compared with non-smokers. It is known [7] that ghrelin levels are also higher in smokers vs non smokers [8], and that these levels are decreased after smoking cessation [9]. Regarding the relation between pack-years and obestatin plasma levels in smokers, we did not find any. This is in accordance also with the findings of Fagerberg et al study [7], where also ghrelin plasma levels were not associated with cigarette years.

Serum obestatin levels did not change upon fasting or feeding in rats [4], but in mice, a slight reduction was seen upon fasting [10]. In humans also 7 no postprandial changes in serum obestatin levels have been noted [11]. For a peptide proposed to affect food intake acutely, this lack of change in circulating concentrations postprandially is surprising [7]. It is important in the future to take into account the ratio of ghrelin to obestatin, and not only absolute values of each peptide. This kind of consideration has been recently used in the evaluation of obesity [8], where the ratio of circulating ghrelin to obestatin was found to be increased. It is possible, that the increased levels of both ghrelin and obestatin in smokers reflect, at least partially, their different status of appetite.

In conclusion, our study showed that there is no acute response of plasma obestatin levels after smoking initiation, although in smokers the levels of plasma obestatin are higher, but not significantly, comparing to non-smokers.

## Competing interests

There were not any funding sources for this research. There are no competing interests of any of the authors of this manuscript.

## Authors' contributions

AK, MF and ST making the primary contribution and holding primary responsibility for the data. MF and DB make the concepts and the interpretation of the results. AT, NK and MS enrolled patients and collected all specimens; they have made also substantial scientific contributions to the study. All authors read and approved the final manuscript
